# Daily Dynamics of Teachers’ Organizational Citizenship Behavior: Social and Emotional Antecedents and Outcomes

**DOI:** 10.3389/fpsyg.2019.02863

**Published:** 2019-12-19

**Authors:** Shiri Lavy

**Affiliations:** Department for Leadership and Policy in Education, University of Haifa, Haifa, Israel

**Keywords:** organizational citizenship behavior, teachers, emotions, supervisor support, colleague support, daily survey

## Abstract

Organizational citizenship behavior (OCB) is considered vital for organizations’ performance, and there is notable interest in factors that foster it. However, recent research has questioned the absolute positivity of OCB and pointed to its understudied possible adverse effects (e.g., on employees’ well-being). The present research aims to shed light on these issues by exploring the daily dynamics of employees’ social and emotional work lives’ interplay with their OCB. Specifically, the research focuses on teachers, whose job enables notable OCB and whose performance is profoundly affected by it. Based on the literature linking work relationships and emotional experiences with OCB, we examined the interplay between teachers’ OCB and their daily perceived supervisor and colleague support, and daily positive and negative emotional experiences. Sixty teachers completed self-report questionnaires of the research variables every day for 12 workdays. Results indicated significant associations of daily supervisor support and negative emotions with increased OCB on the following day, links of daily positive emotions with decreased OCB on the following day, and associations of daily OCB with increased negative emotions on the following day. These findings point to daily processes that may underlie longer term effects of OCB (such as burnout), including a potential downward spiral of negative emotions that seem to result from OCB and reinforce it.

## Introduction

Recent years have seen a notable increase in the study of organizational citizenship behavior (OCB), after research has consistently shown its associations with a host of positive organizational outcomes, including various aspects of organizational effectiveness (Podsakoff et al., 2009). OCB generally refers to employees’ extra-role activities, typically representing “individual behavior that is discretionary, not directly or explicitly recognized by the formal reward system, and in the aggregate promotes the efficient and effective functioning of the organization” (Organ, 1988, p. 4). Recent definitions of OCB are broad, and may even include all performance that supports the social and psychological environment of the organization/employees (Organ, 1997). However, a common theme in most definitions is that in their essence, OCBs are behaviors that go beyond the employees’ official job description and include acts such as helping others, taking on additional responsibilities, and promoting initiatives (Organ et al., 2006; Bolino et al., 2013). As compellingly demonstrated in a meta-analysis of 168 independent samples, OCB was positively associated with increased managerial ratings of employee performance, productivity, efficiency, and customer satisfaction, reduced costs, and decreased employee turnover intentions, turnover, and absenteeism (Podsakoff et al., 2009). It was also argued to facilitate a positive working environment, which enables organizations to attract and retain employees (Organ, 1988; Organ et al., 2006) and increase employees’ positive attitudes toward their job and the organization (e.g., Koopman et al., 2016).

However, in the last decade, some researchers began questioning the absolute positivity of OCB and called for a more balanced examination of OCB – including its effects and antecedents (Bolino et al., 2004). They argued that although OCB may have positive outcomes, OCBs may also have costs that should be realized and more complex antecedents that should be explored. Research that followed this call indeed showed that OCB was associated with some undesirable costs for individuals who perform them: it was related to more work-family conflict (Halbesleben et al., 2009) and less job satisfaction among individuals with low levels of optimism (Munyon et al., 2010). In addition, individual initiative, a specific type of OCB, was also related to role overload and job stress (Bolino and Turnley, 2005). Furthermore, researchers have shown that some OCBs are not performed voluntarily but instead encouraged by supervisors’ expectations or organizational imperatives or politics (Van Dyne and Ellis, 2004; Vigoda-Gadot, 2006). These “compulsory” OCBs are related to job stress, intentions to quit, work-leisure conflict, burnout, and decreased job satisfaction and in-role performance (Vigoda-Gadot, 2007; Bolino et al., 2010). This evidence suggests that a more nuanced exploration of OCB and its potential antecedents and effects is needed, which would deepen our understanding of its positive aspects, as well as its potential costs (Bolino et al., 2013). Thus, the present study aims to provide a more balanced examination of the daily dynamics of OCB among teachers.

### Teachers’ Organizational Citizenship Behavior

Teaching is among the professions in which OCB is essential. Teachers’ and schools’ success fundamentally depend on teachers’ commitment to the school’s goals and values (Somech and Ron, 2007; Somech and Oplatka, 2014) and their willingness to “go above and beyond the call of duty to contribute to successful change,” and schools “cannot anticipate through formally stated in-role job descriptions the entire array of behaviors needed for achieving (their) goals” (Belogolovsky and Somech, 2010, p. 914). Teachers’ OCBs comprise a broad range of activities related to helping behaviors extended to colleagues, supervisors, and students (including special preparations for students at different levels) and contributing to the school at large (e.g., suggesting changes and improvements and advocating for the school) (Somech and Drach-Zahavy, 2000). Most of these behaviors happen daily and are crucial for school functioning. Despite their importance, OCB’s daily antecedents have not been explored to date. Furthermore, teachers’ burnout and decreased well-being are significant concerns in contemporary education research (Ghanizadeh and Jahedizadeh, 2015), and there is concerning evidence pointing to the high prevalence of teacher burnout (Schaufeli and Buunk, 2003; Hakanen et al., 2006) and its troubling effects on teacher turnover and diminished performance (Shen et al., 2015; Grant, 2017). Thus, examining potential adverse effects of teachers’ OCB on their well-being and exploring related daily dynamics is of interest not only for work and organization researchers but also for education researchers, educators, and policymakers.

In the present study, we focus on two aspects that have been closely linked with several indicators of performance/functioning and well-being of employees in general and teachers specifically – social support and emotions. For both, we suggest that it is important to explore their impact on teachers’ OCB but also to examine how they are changed by OCB, exploring potential cyclic effects (initially suggested in a previous study; Halbesleben and Wheeler, 2015). Social support was shown to be an antecedent of OCB, as it contributes to employees’ motivation to go beyond their formal job requirements (Organ et al., 2006); but employees’ OCB can also contribute to supervisors’ and colleagues’ motivation to support the employees and enhance their connection with them. In a similar vein, emotions were typically studied as outcomes of OCB (e.g., Koopman et al., 2016), but they may also impact employees’ tendency to conduct OCBs. Thus, for both factors (social support and daily emotions), we explore potential cyclic effects.

### Social Support

Supportive work relationships were repeatedly associated with positive work-related outcomes such as employees’ satisfaction, well-being, development, and performance (e.g., Dutton and Heaphy, 2003; Cross and Cummings, 2004;Bakker et al., 2005;Chiaburu and Harrison, 2008). Such relationships are typically studied in the context of support from supervisors and from colleagues, who are usually those intimately aware of employees’ tasks, present in their work environment, and often share their tasks, responsibilities, and work challenges. This is also the case for teachers (e.g., Hoy and DiPaola, 2010). Specifically, supervisor support has been associated with increased job satisfaction (e.g., Rhoades and Eisenberger, 2002; Gagnon and Michael, 2004), affective commitment (Casper et al., 2011), and performance (Gagnon and Michael, 2004) and with decreased cynicism (Cole et al., 2006), and turnover (Eisenberger et al., 2002; Smith, 2005; DeConinck and Johnson, 2009). In schools, principals were considered to be “the heart” of the school (Oplatka, 2015), and their supportive leadership is viewed as a key mechanism for building healthy schools, which seek and foster improvement (Hoy and Tarter, 1997; Sebastian and Allensworth, 2012). Similarly, colleague support, received from coworkers who do not have formal authority over one another, was also associated with positive outcomes, such as reduced stress, exhaustion, and burnout (Halbesleben, 2006; Le Blanc et al., 2007) and increased work engagement, performance (Xanthopoulou et al., 2008; Schreurs et al., 2012), and energy (Dutton and Heaphy, 2003). Colleague support was also considered key to schools’ success (Hoy and Tarter, 1997; Hoy, 2012).

Both supervisor support and colleague support were suggested as factors that contribute to employees’ OCB, as they foster employees’ motivation to go beyond their formal job requirements and contribute to specific others in the organization and to the organization itself and boost employees’ resources – which enables them to do so. Supervisor support was highly associated with extra-role performance (Shanock and Eisenberger, 2006), and encouraging leadership was one of the main predictors of OCB found in a broad review of the field (Organ et al., 2006). Furthermore, leadership styles that comprise a strong element of supporting followers, such as ethical leadership and servant leadership, were suggested as an antecedent of fluid internal workplace relationships and group social capital (Linuesa-Langreo et al., 2018). These same styles were repeatedly associated with various positive organizational outcomes, including OCB (e.g., Ruiz-Palomino et al., 2011; Shin, 2012; Linuesa-Langreo et al., 2018). Similarly, colleague support and trust were associated with employee OCB (Singh and Srivastava, 2009; Halbesleben and Wheeler, 2015).

However, these effects have not been thoroughly examined in teachers (despite the importance of OCB for teachers). Furthermore, most of the effects comprise correlational findings, based on cross-sectional data, which cannot infer causal effects. Based on studies that have shown that although social support has a general/stable component, it also has a dynamic component that varies daily, and that fluctuations in this component have notable effects on employees’ behavior (Lavy et al., 2017) including OCB (Halbesleben and Wheeler, 2015), I suggest that:

H1: Daily supervisor and colleague support will be associated with increased teacher OCB on the following day.

Initial research also suggested possible cyclic effects of OCB on relationships with supervisors and colleagues and on their support. Specifically, OCB was suggested as a strategy for receiving more appreciation and support from a supervisor (e.g., Halbesleben and Wheeler, 2015), and colleague OCB was argued to be affected by individuals’ motivation to receive more support from colleagues and increase the sense of communion (Halbesleben and Bowler, 2007); in one study, it was even shown to be associated with previous-day OCB (Halbesleben and Wheeler, 2015). Thus, we explored such cyclic effects, reflecting the effectiveness of OCB in increasing daily social support for teachers.

H2: Daily teacher OCB will be associated with subsequent-day support from the supervisor and from colleagues.

### Emotion and Organizational Citizenship Behavior

Emotions are critically affected by employees’ work experiences, and also affect employees’ behavior and well-being in multiple ways (Ashkanasy, 2002). Over time, experiencing more positive emotions was generally associated with better health (Fredrickson and Levenson, 1998;Danner et al., 2001) and increased work performance, and also with fewer withdrawal behaviors and turnover intentions (Lyubomirsky et al., 2005). Conversely, chronic experiences of negative emotions were associated with health problems (Engebretson et al., 1989), emotional exhaustion (Brotheridge and Grandey, 2002), and decreased work performance (Wright and Cropanzano, 1998).

A growing body of evidence suggests that prosocial behavior (in various life arenas) promotes positive emotional rewards for the giver. For example, studies have documented links between volunteerism (Thoits and Hewitt, 2001; Borgonovi, 2008), helping (Post, 2011) and generous behavior (Dunn et al., 2008; Aknin et al., 2015), and positive emotions and well-being. Specifically among teachers, the feeling that their work contributes to others was shown to increase their positive relationships with others, which in turn increased their job satisfaction (Lavy and Bocker, 2018). Studies have also specifically linked OCB with positive emotions (e.g., Dalal et al., 2009; Lavy and Littman-Ovadia, 2017) and have even demonstrated that engaging in OCBs predicted subsequent positive affect (Glomb et al., 2011; Koopman et al., 2016), as expected from voluntary behavior that contributes to others and is expected to yield feelings of fulfillment and meaningfulness. However, certain studies suggest that OCBs may sometimes be driven by organizational politics or supervisor expectations, and not by prosocial motives (Van Dyne and Ellis, 2004; Vigoda-Gadot, 2006), in which cases they are associated with negative emotional experiences such as stress, burnout, and decreased job satisfaction (Vigoda-Gadot, 2007; Bolino et al., 2010).

While acknowledging the different lines of evidence, which suggest that OCB may lead to positive or negative emotional experiences, in the present study, we aimed to shed light on the daily effects of teacher OCB on their emotional states, by openly exploring these two possibilities.

H3: Daily teacher OCB will be associated with a change in positive and negative emotions on the following day. Alternative a – positive emotions will increase and negative emotions will decrease. Alternative b – negative emotions will increase and positive emotions will decrease.

Furthermore, research suggests that emotions can impact subsequent behavior, with potential cyclic effects on OCB. Research on positive emotions suggests that they contribute to prosocial behaviors of employees and leaders (Michie, 2009; Cavanaugh et al., 2015), and one study has specifically demonstrated that positive emotions elicited by prosocial behaviors can increase subsequent prosocial behavior (Aknin et al., 2012). However, as OCB may be related to other motives (which are not necessarily prosocial), potential cyclic effects may be different. Initial studies have shown mixed results regarding these potential effects while indicating associations of employees’ emotional experiences with their OCBs. Trougakos et al. (2015) showed that feeling drained was associated with decreased subsequent OCB, and Glomb et al. (2011) showed that negative emotions predicted more subsequent altruism OCBs (presumably because these OCBs are used for mood repair), while positive emotions were not associated with subsequent OCBs. Additional examination of the effects of emotions on OCB is required in order to understand them fully. The present study provides the required exploration of these issues and attempts to openly explore the potential effects of teachers’ negative and positive emotions on their daily OCB.

H4: Daily teachers’ emotions will be associated with a change in OCB on a subsequent day.

### The Present Study

**Figure 1:**
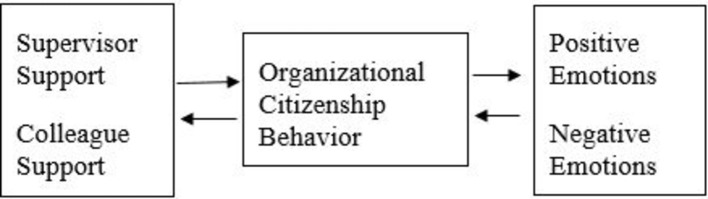
Integrative theoretical model of the hypothesized daily dynamics.

The present study aimed to explore the research hypotheses and questions (summarized in Figure 1) in a daily-diary survey, which enables linking daily fluctuations in a certain variable with daily changes in another variable on a subsequent day. Such links may imply that the fluctuations in the second variable derive, to some extent, from the changes in the first variable (which precede them) (e.g., Xanthopoulou et al., 2008; Lavy et al., 2014). This method can be used when the researched variables demonstrate notable daily fluctuations. Research has shown that this is indeed the case for social support (Lavy et al., 2017), emotions (Yang and Diefendorff, 2009) and OCB (Halbesleben and Wheeler, 2015), and thus a daily survey method can be helpful in examining their daily dynamics and potential daily effects on each other.

## Method

### Participants

The study comprised 60 Israeli teacher participants, in line with the recommended sample size for multilevel modeling of this kind (Maas and Hox, 2005) and used in similar daily-diary studies (e.g., Lavy et al., 2013; Lavy and Eshet, 2018). Most participants were women (74%), corresponding with the majority of women teaching in Israel. They taught in high (47%), middle (15.2%), and elementary (37.8%) schools; their ages were 24–64 (*M*_age_ = 39.32, SD_age_ = 10.36); and their tenure was 1–38 years (*M*_tenure_ = 13.27, SD_tenure_ = 10.63). The teachers were Jewish (63.6%), Muslim (25.8%), Druze (6.1%), or Christian (4.5%) and had a bachelor’s degree (62.1%), master’s degree (36.4%), or another secondary-education diploma (1.5%).

### Measures

We used shortened versions of all the measures to avoid fatigue effects due to repeated completion of the daily survey. The items were chosen based on their loadings on the original scales and their relevance to teachers’ daily experiences. Table 1 presents the scales’ means, standard deviations, reliabilities, and ICCs.

**Table 1:** Means, standard deviations, reliabilities ICCs, and zero-order correlations among the study daily variables, across all the study days.

	Means	SD	Reliability (Chronbach’s *α*)	ICC	1	2	3	4
1. OCB	2.95	2.11	0.91	0.75				
2. Supervisor support	2.68	2.26	0.96	0.71	0.73^***^			
3. Colleague support	3.90	2.54	0.97	0.80	0.76^***^	0.71^***^		
4. Positive emotions	5.05	1.33	0.89	0.28	0.20^***^	0.29^***^	0.20^***^	
5. Negative emotions	2.25	1.28	0.79	0.31	0.14^***^	−0.01	0.02	−0.31^***^

*** *p < 0.001*.

#### Daily Teacher Organizational Citizenship Behavior

Daily teacher OCB was assessed with a shortened five-item version of the teacher OCB measure (Somech and Drach-Zahavy, 2000). Teachers ranked on a scale of 1 (not at all) to 7 (to a great extent) the extent to which they participated in actions described in each item during the day (e.g., “Today I took responsibility on or volunteered to do something that is not an integral part of my job”; “Today I helped a colleague or the principal”).

#### Daily Supervisor Support

Daily Supervisor Support was assessed with a shortened three-item version of the Perceived Supervisor Support measure (Eisenberger et al., 2002), modified to describe daily experiences. Items (e.g., “Today my supervisor went out of his/her way to help me at work”) were ranked on a 1 (not at all) to 7 (to a great extent) scale.

#### Daily Colleague Support

Daily Colleague Support was assessed with a brief two-item measure (“Today I felt that my colleagues at work support me”; “Today I received social-emotional support from my colleagues”), ranked on a 1 (not at all) to 7 (to a great extent) scale.

#### Positive and Negative Affect

Positive and Negative Affect were assessed with six items, including positive (*enthusiastic, happy, proud*) and negative (*stressed, nervous, guilty*) items from the Positive and Negative Affect Schedule (PANAS; Watson et al., 1988; Watson and Clark, 1999). Participants ranked the extent to which they felt each emotion during their workday on a 1 (not at all) to 7 (to a great extent) scale.

### Procedure

After receiving approval from the University of Haifa Faculty of Education Ethics Committee (approval number 074/17), students in a research seminar were invited to contact two to five teachers and invite them to participate in the study voluntarily (with no monetary compensation). Those who expressed interest received a brief overview of the study and, if they agreed to participate, signed a consent form and completed a demographic questionnaire. Then, participants received daily prompts to complete a brief online survey every day (excluding weekends) for 12 workdays. Participants who missed the survey completion for 1–3 days received additional prompts to complete the survey at the end of the 12 days. Those who missed more than 4 days altogether were dismissed from the study. Participants’ attrition rate was ~30%.

### Data Analysis

After initial examination of the variables’ reliabilities, means, and standard deviations, we computed variables’ interclass correlations (ICCs) to examine the need for Hierarchical Linear Modeling (HLM). The ICCs ranged from 0.28 to 0.80 (Table 1), indicating daily variability within teachers, justifying a multilevel analysis (Raudenbush and Bryk, 2002). The day-level variables (level 1) were nested within the teacher-level (level 2), thus accounting for the joint variance of responses of the same teacher on different days. The dependent variable comprised its daily rating, and the independent variable included mean ratings of the predicting variable on the previous day. In addition, the independent variable’s ratings on the previous day were controlled (they were entered as another independent variable), to enable predicting the associations of previous-day ratings of the independent variable with the *change* in the dependent variable ratings on the following day. Teacher-level variables (teaching load, tenure, and gender) were also controlled. Cyclic effects were examined by conducting a similar analysis, in which the independent and dependent variables were exchanged, as done in previous studies (e.g., Lavy and Eshet, 2018). The variables were entered into the equation uncentered, to maintain scale consistency across the independent and dependent variables. This methodology is common in daily-diary surveys analysis (e.g., Lavy et al., 2017).

## Results

The HLM analyses (Table 2) partially supported H1, while indicating that supervisor support (but not colleague support) on a specific day was associated with increased teacher OCB on the following day (*b* = 0.11, *p* < 0.05). There were no cyclic effects to social support (H2), as OCB was not associated with subsequent changes in daily perceived social support.

**Table 2:** HLM coefficients predicting daily fluctuations in teachers’ daily OCB, social support, and positive and negative affect.

	**Predicting daily OCB from social support**					
	**Predicting OCB from PD supervisor support**			**Predicting OCB from PD colleagues’ support**		
	**Coefficient**	**SE**	*T*	**Coefficient**	**SE**	*T*
PD OCB (control)	0.13^*^	0.06	2.42	0.19^***^	0.05	3.60
PD supervisor support	0.11^*^	0.05	2.15			
PD colleagues’ support				−0.02	0.05	−0.37
^Teaching load	0.05	0.04	1.31	0.05	0.04	1.38
^Tenure	−0.02	0.02	−1.11	−0.03	0.02	−1.23
^Gender	0.46	0.47	0.98	0.52	0.50	1.05
	**Potential daily OCB outcomes**					
	**Predicting positive emotions from PD OCB**			**Predicting negative emotions from PD OCB**		
	**Coefficient**	**SE**	*T*	**Coefficient**	**SE**	*T*
PD positive emotions (control)	−0.02	0.05	−0.47			
PD negative emotions (control)				0.17^***^	0.04	3.87
PD OCB	−0.02	0.04	−0.41	0.09^**^	0.03	2.73
^Teaching load	0.05^*^	0.02	2.05	−0.01	0.02	−0.56
^Tenure	−0.00	0.01	−0.28	−0.02	0.01	−1.90
^Gender	0.35	0.29	1.18	−0.10	0.21	−0.48
	**Cyclic effects**					
	**Predicting supervisor support from PD OCB**			**Predicting colleague support from PD OCB**		
	**Coefficient**	**SE**	*T*	**Coefficient**	**SE**	*T*
PD supervisor support (control)	0.11^*^	0.05	2.22			
PD colleagues’ support (control)				0.21^***^	0.05	4.06
PD OCB	0.05	0.06	0.90	0.09	0.04	1.60
^Teaching load	0.06	0.04	1.29	0.07	0.04	1.76
^Tenure	−0.04	0.02	−1.66	−0.03	0.02	−1.57
^Gender	0.55	0.54	1.02	1.18	0.50	2.35
	**Predicting OCB from PD positive emotions**			**Predicting OCB from PD negative emotions**		
	**Coefficient**	**SE**	*T*	**Coefficient**	**SE**	*T*
PD OCB (control)	0.28^***^	0.05	5.56	0.23^***^	0.05	4.90
PD positive emotions	−0.22^***^	0.05	−3.94			
PD negative emotions				0.16^**^	0.06	2.88
^Teaching load	0.06	0.04	1.58	0.05	0.04	1.45
^Tenure	−0.02	0.02	−1.20	−0.02	0.02	−1.04
^Gender	0.48	0.45	1.07	0.46	0.45	1.02

^ = *Level 2 variables*.

* p < 0.05;

** p < 0.01;

*** *p < 0.001*.

OCB, organizational citizenship behavior; PD, previous day; significant coefficients of independent variables (not control variables) are underlined.

The analysis provided partial support for H3 – indicating a significant association of daily OCB with increased negative emotions on the following day (*b* = 0.09, *p* < 0.01). However, OCB had no significant effect on positive emotions. Examination of the potential cyclic effects (H4) showed that positive emotions were associated with decreased OCB (*b* = −0.22, *p* < 0.001) and negative emotions were associated with increased OCB (*b* = 0.16, *p* < 0.01) on the following day.

## Discussion

**Figure 2:**
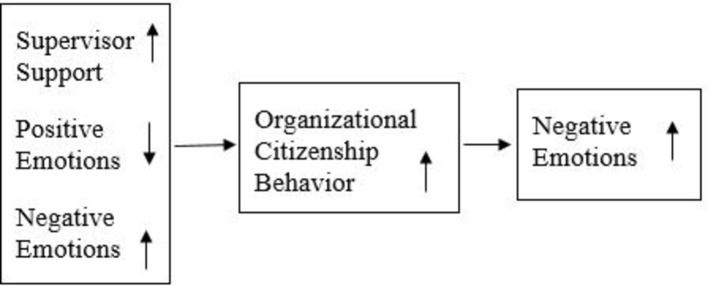
The updated integrative model (based on a conceptual summary of the findings).

The present study aimed to provide a balanced exploration of the daily dynamics of teachers’ OCB with social support and emotions. Examination of direct and cyclic effects of the variables revealed significant associations of daily supervisor support and negative emotions with increased OCB on the following day, links of daily positive emotions with decreased OCB on the following day, and associations of daily OCB with increased negative emotions on the following day (Figure 2). These findings shed light on daily processes that may underlie some of the longer term effects that were discussed in the context of OCB and have theoretical and practical implications.

### Daily Social Support and Organizational Citizenship Behavior

The findings suggest that supervisor support is qualitatively different from colleague support, and more important in the context of OCB. Daily supervisor support seems to serve as a notable daily antecedent of OCB (in correspondence with previous findings; Organ et al., 2006), while colleague support does not. This unique impact of supervisors’ support on their employees’ behaviors and attitudes was also evident in other studies (e.g., Lavy, 2014; Lavy et al., 2017). Practically, these findings suggest that daily supervisor support may be an effective mechanism for encouraging teacher OCBs. Thus, it may be worth investing the time and effort of school leadership in this kind of support, as it may “pay off” in eliciting teachers’ contributions to the school.

The findings also suggest no significant effects of daily OCB on perceived daily supervisor support nor on colleague support (beyond the effects of teaching load, tenure, and gender). These results may suggest that OCBs can contribute to social support over longer time periods – as it takes time before they are acknowledged. But they may also suggest that the actual benefits of OCBs to employees may be limited (at least at the daily level) – not yielding increased social support in the short term.

### Daily Emotions and Organizational Citizenship Behavior

In the attempt to provide a balanced examination of the daily effects of OCB on teachers’ emotions in the present study, the negative effects of OCB proved to be significant, while the positive effects were not: daily OCB was associated with a subsequent increase in negative emotions and was not significantly associated with a change in positive emotions. This finding corresponds with literature suggesting that OCB can enhance negative feelings like stress and exhaustion (e.g., Bolino et al., 2013). The different patterns for positive and negative emotions have also been demonstrated in previous research on teachers’ emotional experiences (e.g., Lavy and Eshet, 2018) and may reflect the bias and higher intensity of negative emotional reactions (e.g., Fredrickson, 2013).

Furthermore, the findings interestingly point to the significant effect of daily emotions on subsequent OCB, indicating that emotional experiences may be important antecedents of OCB. In line with previous findings (Glomb et al., 2011), negative emotions were associated with increased OCB on the following day, and, in addition, positive emotions were associated with decreased OCB on the following day. This interesting finding suggests that when teachers are happy and feeling good, they are less inclined to engage in OCBs on the following day. However, and this is a finding that may reflect a concerning process, teachers tend to use OCB as a mood repair mechanism (as suggested by Glomb et al., 2011). Unfortunately, this mood repair mechanism seems to be unsuccessful, as OCB is associated with an additional subsequent increase in negative emotions, a process that may trigger a downward spiral of increasing negative affect. Over time, this process may result in depletion of emotional resources, exhaustion, and burnout – outcomes that were indeed suggested in previous critiques of OCB (e.g., Bolino et al., 2013). Beyond the personal cost of such effects for teachers’ well-being, negative emotions also impact teachers’ work and limit their ability to function optimally (e.g., Lavy and Eshet, 2018).

The findings point to the emotional costs of teacher OCB and to the need to acknowledge and address them by mitigating their adverse effects, exploring their sources, and repairing their negative impact. One of these ways may be increasing teachers’ positive emotions (in order to balance/overcome the negative emotions), for example, by increasing communion and colleague support – which may also moderate the adverse effects of OCB. Together with the limited effects on teachers’ perceived social support, the findings suggest that the costs of OCB may not be worthy of their limited value for teachers as individuals.

## Limitations and Future Research

The study’s findings should be considered while acknowledging its limitations. The study comprised only self-report data – subjected to bias and social-desirability effects. The sample comprised only Israeli teachers, recruited in convenience sampling. The responses were collected daily throughout 12 workdays – emotional responses vary momentarily, and thus experience sampling may have been more accurate for some of the measures (although such intervals may be less relevant for other study variables). It would be beneficial to explore the longer term implications of the study’s findings (such as potential cumulative effects on burnout), and to expand this kind of research to additional professions and populations. It would also be helpful to explore additional factors that may affect OCB and be affected by them and to explore potential organizational and individual moderators of such processes (such as the previously suggested factors of organizational culture and personality). This kind of research can promote an integrative understanding of the complex dynamics related to OCB.

## Data Availability Statement

The datasets generated for this study are available on request to the corresponding author.

## Ethics Statement

The studies involving human participants were reviewed and approved by The University of Haifa Faculty of Education Ethics Committee. The participants provided their written informed consent to participate in this study.

## Author Contributions

The author confirms being the sole contributor of this work and has approved it for publication.

### Conflict of Interest

The author declares that the research was conducted in the absence of any commercial or financial relationships that could be construed as a potential conflict of interest.
